# Spatiotemporal disparities in the diagnosis, treatment, and mortality of respiratory tract cancers in Brazil

**DOI:** 10.1371/journal.pone.0334115

**Published:** 2025-11-12

**Authors:** Miyoko Massago, Vlaudimir Dias Marques, Gustavo Cezar Wagner Leandro, Matheus Henrique Arruda Beltrame, Rogério do Lago Franco, Mamoru Massago, Júlia Kaori Uguma Mizuta, Celso Ivam Conegero, Samile Bonfim, Vinícius Lopes Giacomin, Sanderland José Tavares Gurgel, Oscar Kenji Nihei, Maria Dalva de Barros Carvalho, Luciano de Andrade

**Affiliations:** 1 Health Sciences Department, State University of Maringa, Maringa, Parana, Brazil; 2 Medicine Department, State University of Maringá, Maringa, Parana, Brazil; 3 Department of Computer Sciences, State University of Maringa, Maringa, Parana, Brazil; 4 Pharmacy Department, State University of Maringá, Maringa, Parana, Brazil; 5 Morphological Sciences Department, State University of Maringá, Maringa, Parana, Brazil; 6 Center of Education, Literature and Health, Western Parana State University, Foz do Iguaçu, Parana, Brazil; Saint Mary's University, CANADA

## Abstract

**Purpose:**

This study aimed to assess spatiotemporal disparities in the diagnosis, treatment, and mortality rates of respiratory tract cancers (RTCs) in Brazil.

**Methods:**

Data on 40–79-year-old Brazilian patients diagnosed with RTCs between 2013 and 2022 were analyzed using spatial and temporal analysis. We also calculated the healthcare and mortality ratio (HMR), defined as the relationship between diagnosis/treatment density and mortality, to provide an additional measure of healthcare disparities.

**Results:**

Space-time cubes analysis (STC) revealed significant increasing trends in diagnosis (trend statistic = 3.22, *p* = 0.0013) and treatment (trend statistic = 3.04, *p* = 0.0042) and stable trends in mortality (trend statistic <0.001, *p* = 1.0000), with hot spots in the South and Southeast and cold spots in the Northeast for diagnosis and treatment, whereas mortality displayed persistent high values in the South, with more variable patterns across other regions. Temporal graph analysis showed that the analyzed variables peaked in 2019 and decreased in the following year. It was also observed that, from 2013–2017–2018–2022, diagnosis and treatment rates expanded from the South to the Southeast and Central-West, whereas mortality did not differ significantly between the two quinquennia. Global spatial autocorrelation (Moran’s *I* > 0.85) was observed for diagnosis, treatment, and mortality rates in both quinquennia. Univariate local indicators of spatial association analysis identified high–high clusters for diagnosis, treatment and mortality, mainly in the South, and low–low clusters in the North and Northeast. In 2013–2017, high HMR values were limited to northern Parana state, the southwestern of Mato Grosso do Sul state, the southern portion of Goias state, and western area of Minas Gerais. In the next quinquennia, HMR improved across almost all the South and Southeast regions and expanded into parts of Center-West, North, and Northeast.

**Conclusion:**

The observed improvements in diagnostic and treatment services have not resulted in a consistent and widespread reduction in RTC mortality across the country, despite recent efforts to expand service delivery beyond major urban centers.

## Introduction

Respiratory tract cancers (RTCs) are among the deadliest types of cancers, representing a serious public health challenge worldwide. Globally, 2,669,866 cases of RTCs were recorded in 2020, resulting in 1,920,828 deaths in the same year. In Latin America, 122,633 RTC cases and 101,319 deaths were recorded in 2022, with Brazil accounting for 53,080 cases and 43,803 deaths [1]. Although advanced-stage RTCs have a high mortality rate, early detection remains crucial for more effective and less invasive therapeutic interventions, considerably improving the chances of cure and enhancing quality of life [2].

Brazil’s 2021–2030 Strategic Action Plan for Tackling Chronic and Noncommunicable Diseases outlines several health promotion strategies and actions [3]. Some examples include awareness campaigns on protective factors for cancers, investment in survey-based research to improve our understanding of diseases and their risk factors, and the development of control programs to reduce cancer incidence, prevalence, and mortality in the country [3].

Nonetheless, it is important to highlight that Brazil faces significant regional disparities in healthcare, owing to its vast territorial extension and marked differences in socioeconomic development. These factors, coupled with the heterogeneity in the distribution of oncology services, may result in delayed diagnosis and treatment, hindering the implementation of control strategies for noncommunicable diseases [3–4]. Thus, it is imperative to improve data collection and cancer surveillance for the population impact assessment and the implementation of effective prevention and control programs, including quality assessments for diagnostic services and screening tests [3].

Previous studies showed an uneven distribution of cancer incidence across Brazil. This disparity is likely due to differences in access to advanced diagnostic services and specialized treatments, which are more readily available in urban and developed areas [5–9]. Mortality from RTCs may also be affected by these inequalities, reflecting potential regional and temporal variations resulting from the complex interplay of socioeconomic conditions, healthcare access, and risk factors [6,10–12].

Spatial and spatiotemporal analyses are mathematical modeling methods used to detect geographic patterns and assess the distribution of events over time. These approaches have been increasingly applied in oncology to investigate disparities in diagnosis, treatment, and mortality, revealing persistent regional inequalities and highlighting areas requiring targeted interventions [12–14]. In addition, in the context of respiratory diseases, such as COVID-19 [15], they have been used to understand health system disruptions and temporal variations. This body of evidence reinforces the importance of spatial and temporal analyses, not only during acute health crises but also in the ongoing surveillance and control of chronic diseases.

In view of the foregoing, this study aimed to analyze the distribution of RTC diagnosis, treatment, and mortality in Brazil between 2013 and 2022, focusing on the identification of spatiotemporal patterns and inequalities.

## Methods

### Study design

This is an ecological, cross-sectional, descriptive, and retrospective study assessing spatiotemporal disparities in RTC diagnosis, treatment, and mortality in Brazil. The protocol follows the Strengthening the Reporting of Observational Studies in Epidemiology (STROBE) statement [16].

Brazil, the largest country in Latin America in terms of territorial dimensions, has a total area of 8,515,767.049 km^2^, with an estimated population of 203,080,756 inhabitants in 2022. The per capita gross domestic product was US$7,041.94 in 2023, and the illiteracy rate was estimated at 5.4% in that same year [17]. The country is composed of five administrative regions divided into 26 federative units and the Federal District, encompassing 5568 municipalities and 438 health regions. Health regions are administrative units defined by the Ministry of Health to organize and coordinate healthcare delivery within the Unified Health System (SUS). Each health region consists of contiguous municipalities sharing cultural, economic, and social characteristics, as well as integrated communication networks and transportation infrastructure. In this study, data were initially obtained at the municipal level and subsequently aggregated by health region. This aggregation reduced random fluctuations in small-area rates and aligned the analysis with the organizational structure used for healthcare planning in Brazil [17–18].

### Data sources

This study used secondary data obtained from publicly available sources. The absolute number of Brazilian individuals aged 40–79 years diagnosed with RTCs between 2013 and 2022 was obtained from the Brazilian Oncology Database (*Painel Oncologia*) [20]. RTCs were identified by selecting all cases classified as C32 (malignant neoplasm of the larynx), C33 (malignant neoplasm of trachea), or C34 (malignant neoplasm of bronchus and lung), according to the 10^th^ revision of the International Classification of Diseases (ICD-10) [19]. For mortality data, only deaths in which these ICD-10 codes were recorded as the underlying (basic) cause of death on the death certificate were included. Information on the number of patients treated for these conditions was also obtained from the Oncology Database.

The absolute number of deaths from RTCs in the same age group, as well as the year and locality of each occurrence, was obtained from the Mortality Information System, a platform maintained by the Department of Informatics of the Brazilian Unified Health System (DATASUS) [20]. Data on the 40- to 79-year-old resident population and the cartographic database of each health region and each municipality were obtained from the Brazilian Institute of Geography and Statistics [21–22].

All data were collected between June 30 and August 1, 2024. Data analysis was performed between July 1 and December 30, 2024.

The datasets are publicly available and already anonymized by the data providers before release, containing no identifiable personal information (e.g., names, addresses or document numbers). In addition, the data are aggregated at the municipal or regional level, which ensures patient confidentiality of location. Therefore, no additional anonymization or aggregation was required on our part.

### Rate smoothing

To reduce the instability in regions with small populations, smoothed diagnosis, treatment, and mortality rates were calculated using spatial empirical Bayes smoothing implemented in GeoDa, version 1.12.0 [23]. First, data were downloaded at the municipal-level and then aggregated to the average values of each health region, which are administrative units defined by the Brazilian Ministry of Health to coordinate healthcare services across municipalities. Raw rates were then calculated by dividing the number of diagnoses, treatments, and deaths due to RTCs by the population at risk in each health region, limited to individuals aged 40–79 years. This age restriction served as a method of standardization, focusing on a population with a high incidence and mortality of RTCs and ensuring comparability across regions and time periods. To define the spatial neighborhood relations, a queen-type contiguity neighborhood matrix was applied. Finally, the smoothed rates were then expressed per 100,000 population [24].

### Space–time cube (STC) and emerging hot spot analysis

Spatiotemporal trends in smoothed rates of RTC diagnosis, treatment, and mortality between 2013 and 2022 in Brazil were assessed using STC and emerging hot spot analyses in ArcGIS Pro, version 3.0.36056 [25]. STC analysis organizes information into a three-dimensional structure, where the horizontal axes represent geographical locations (health regions) and the vertical axis corresponds to yearly time intervals [25].

A shapefile and a database containing health region codes and smoothed rates for each year were used to generate a cube formed by space–time bins. This step was performed using the tool “Create space–time cube from defined locations” Each bin within the cube stores aggregated values, such as counts, averages, or sums by location and period, corresponding to a specific health region and year. Spatiotemporal trends were evaluated through the Mann–Kendall trend test applied to each space–time cube bin, using ArcGIS Pro for statistical implementation [25]. This approach facilitated the detection of meaningful spatiotemporal patterns, including emerging trends, temporary peaks, and cyclical variations. Following the creation of the STC, emerging hot spot analysis was performed using ArcGIS Pro [26]. This technique applies statistical methods to identify locations where rates consistently increase or decrease over time. Warm colors (e.g., red) indicate hot spots (areas with high and/or increasing rates), whereas cold colors (e.g., blue) represent cold spots (areas with low and/or decreasing rates). Based on temporal consistency and intensity, hot spots and cold spots were categorized as persistent, sporadic, emerging, or diminishing [26].

### Temporal analysis

As a complement to spatiotemporal analysis, temporal trends were examined by generating line graphs of smoothed rates stratified by administrative region, defined as the five official geographic regions of Brazil (North, Northeast, Southeast, South, and Center-West). This approach facilitated the identification of patterns, peaks, and declines over time, offering a detailed view of trends in RTC diagnosis, treatment, and mortality.

Data were stratified by administrative region and analyzed using the Mann–Kendall trend test to assess the statistical robustness of visual observations. This non-parametric method is commonly used to evaluate trends in time series without assuming a specific distribution [27] and was implemented in R software, version 3.6.0 [28], with RStudio, version 4.3.2 [29], using the Kendall package.

### Analysis of spatial patterns

Spatial autocorrelation analysis was performed to assess the degree of similarity between health regions and refine the results of spatial analysis. Autocorrelation was examined for the mean smoothed rates aggregated into two quinquennia (2013–2017 and 2018–2022). The use of five-year periods allowed for more stable rate estimates by reducing annual fluctuations and provided a structured comparison of medium-term changes in cancer care and mortality in Brazil, where substantial shifts in health policies and service delivery may occur within a decade. Comparisons between groups were conducted using the Wilcoxon–Mann–Whitney test [30].

The global spatial autocorrelation index (Moran’s *I*) and local indicators of spatial association (LISA) were calculated in GeoDa software, version 1.12.0 [23]. Moran’s *I* ranges from −1–1, with values equal to zero indicating spatial randomness (lack of autocorrelation). Negative values indicate negative spatial autocorrelation (i.e., dissimilar neighboring values), which becomes stronger as values approach −1. Positive values indicate positive spatial autocorrelation (i.e., similar neighboring values), which becomes stronger as values approach 1 [32]. Although Moran’s *I* provides an overall measure of spatial clustering across the entire study area, it does not detect local clusters or spatial heterogeneity [31]. Therefore, LISA analysis was conducted to identify specific spatial clusters and spatial outliers. LISA classifies clusters into four categories: high–high (health regions with high rates surrounded by neighbors with similarly high rates), low–low (health regions with low rates surrounded by neighbors with low rates), high–low (health regions with high rates surrounded by neighbors with low rates), and low–high (health regions with low rates surrounded by neighbors with high rates) [32]. Choropleth maps were created to illustrate the spatial distribution of clusters using QGIS version 3.22.5 [33].

### Healthcare and mortality ratio (HMR)

Spatial dynamics relating healthcare efforts (diagnosis and treatment) and mortality outcomes were assessed by constructing a spatial ratio map using weighted kernel density estimation (KDE) techniques and raster analysis in QGIS version 3.22.5 [34–35]. The centroids of the health regions were obtained to represent the spatial anchors of each regional unit, and were linked to the corresponding values of diagnosis, treatment, and mortality rates [36]. For each variable, a weighted KDE was applied, using smoothed mortality rates as weights.

The estimation at location x is given by;


$$\hat{f}(x)\frac{1}{{nh}^{2}}\sum_{i=1}^{n}k\left(\frac{d(x,x(i))}{h}\right)\mathrm{\backslash ]}$$

Where n is the number of observed points, h is the best k bandwidth (set at 470 km), d(x, x(i)) is the Euclidean distance between the estimation point x and the observed point x(i), and k is the Gaussian kernel function.

This approach transformed discrete point data into continuous raster surfaces representing the spatial intensity of each variable while incorporating the spatial influence around each point. The bandwidth was set at 470 km, determined by maximizing the difference between observed and theoretical values of Ripley’s K-function [37], a method used to identify the optimal clustering distance. This value provided stable kernel surfaces while adequately capturing regional differences in spatial distribution, ensuring comparability across both northern (larger distances) and southeastern (shorter distances) regions [38–39].

Subsequently, using raster calculator tools, the KDE surfaces of diagnosis and treatment were summed, producing a layer that represents the combined intensity of healthcare efforts. This combined surface was then divided by the mortality KDE surface, resulting in a spatial ratio raster. To avoid division by zero errors, a constant value of 1 was added to the mortality layer. The resulting index is given by:


$$HMR\;(x)=\frac{KDEdiag(x)+KDEtreat\;(x)}{KDEmort\;(x)+1}\mathrm{\backslash ]}$$

Where KDE diag, KDEtreat and KDEmort represent the kernel density surfaces of diagnosis, treatment, and mortality, respectively, and the constant 1 was added to the denominator to avoid division by zero.

This ratio highlights areas where diagnostic and treatment efforts are high relative to mortality, allowing the identification of regions with better outcomes in controlling mortality. Additionally, it reveals regions where high mortality persists despite considerable healthcare effort, suggesting potential gaps in care delivery or the presence of local risk factors [37–39].

Division by zero errors was avoided by replacing pixels with null values in the mortality layer with minimum substitute values. The raster resolution was adjusted to balance spatial detail and computational efficiency [39]. The resulting raster was visualized through thematic maps, enabling a spatial interpretation of the kernel ratio. These visualizations facilitated the identification of high-risk hotspots and successful regions within the regional healthcare network.

### Ethical approval

The research protocol was approved by the Human Research Ethics Committee at the State University of Maringá, Paraná, Brazil (protocol No. 6,464,116/2023). In accordance with Resolution No 466/2012 of the Brazilian Ministry of Health, and given the nature of the data, the requirement for informed consent was waived.

## Results

Between 2013 and 2022, 140,245 new cases of RTCs were diagnosed in Brazil in individuals aged 40–79 years. During the same period, 119,460 patients were treated and 260,969 died from RTCs. It can be seen from these data that the number of patients treated was 14.80% lower than the total number of diagnosed cases, attributed to ongoing treatments from previous years or limited access to treatment. It is also noteworthy that the number of deaths was 114.78% higher than that of recorded diagnoses, possibly from RTC cases diagnosed in previous years.

The majority (64.50%, 90,466/140,245) of patients diagnosed with RTCs were male. Of the total number of cases, 0.27% (376/140,245) were tracheal cancers, 29.91% (41,951/140,245) were laryngeal cancers, and 69.66% (97,708/140,245) were lung and/or bronchial cancers. Most patients who received treatment for RTCs (65.98%, 78,830/119,460) were male. Among treated patients, 0.12% (146/119,460) had tracheal cancers, 31.16% (37,227/119,460) had laryngeal cancers, and 70.22% (83,885/119,460) had lung and/or bronchial cancers. Most deaths (62.78%, 163,830/260,969) occurred in male patients, with 0.31% (802/260,969) being due to tracheal cancers, 14.94% (38,989/260,969) to laryngeal cancers, and 84.88% (221,519/260,969) to lung and/or bronchial cancers.

### Spatiotemporal analysis

Spatiotemporal analysis of smoothed RTC rates between 2013 and 2022 revealed a significant increase in diagnosis (trend statistic = 3.22, *p* = 0.0013) and treatment (trend statistic = 3.04, *p* = 0.0042), whereas mortality remained stable (trend statistic = 0.00, *p* = 1.0000). This finding indicates that, despite the increase in diagnoses and treatments in some regions of the country, mortality remained high and unaltered.

**Fig 1:**
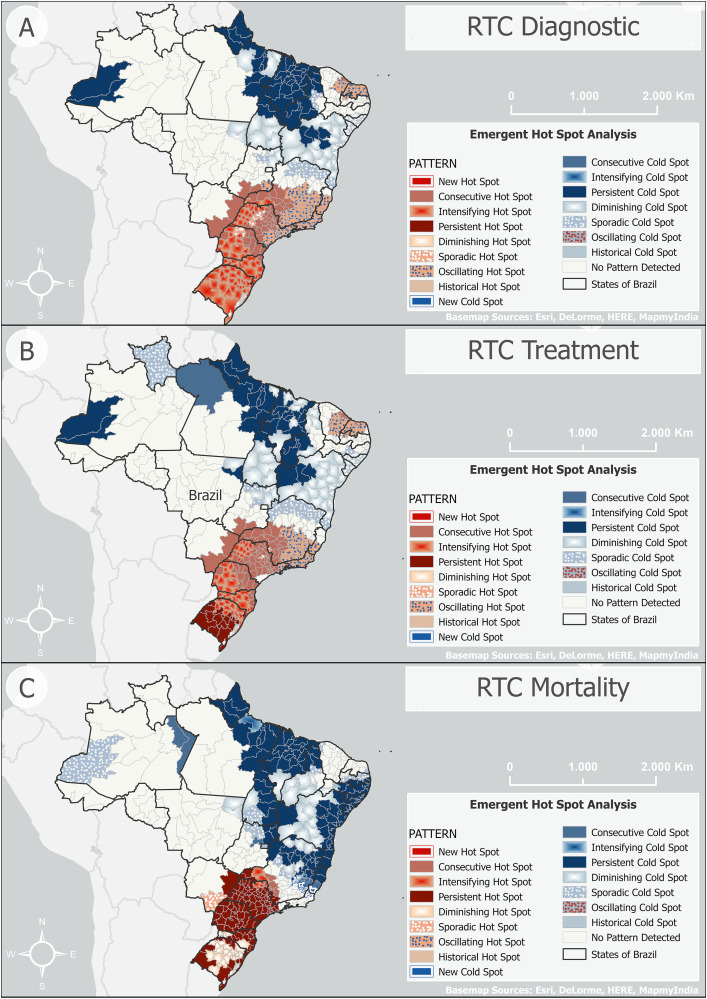
Spatiotemporal trends in the smoothed rates of (A) diagnosis, (B) treatment, and (C) mortality due to respiratory tract cancers between 2013 and 2022 in Brazil, stratified by health region.

Regarding RTC diagnosis rates, the entire South region and a large part of the Southeast exhibited intensifying hot spots, consecutive hot spots, and oscillating hot spots. These patterns extended into transitional zones near the borders of Southeast, South, and Central-West regions, particularly in Southern Goiás, Eastern Mato Grosso do Sul, Western São Paulo, and Southwestern Minas Gerais. Oscillating hot spots were notably present along coastal areas of the Southeast and some parts of the Northeast. Persistent cold spots occurred in the Northeast and North, and diminishing cold spots were observed in the Northeast (Fig 1A). Similar patterns were observed for RTC treatment rates. There were persistent and intensifying hot spots concentrated in the South and Southeast, with the addition of sporadic cold spots in Roraima and part of Minas Gerais State (Fig 1B). For RTC mortality, there was an extensive diminishing hot spot in the South, particularly in Rio Grande do Sul and Santa Catarina States. Persistent hot spots extended throughout Paraná State and transitional areas at the intersection of the South, Southeast, and Central-West regions, including southern Goiás, eastern Mato Grosso do Sul, western São Paulo, and southern Minas Gerais. The Central-West also displayed small sporadic hot spots, mainly in Mato Grosso do Sul. A limited area of the Southeast exhibited intensifying hot spots. In the North, persistent cold spots were surrounded by diminishing and sporadic cold spots, especially across Amazonas, Pará, and parts of Roraima. We also noticed persistent and diminishing cold spots in the Northeast (Fig 1C).

### Temporal analysis

**Fig 2:**
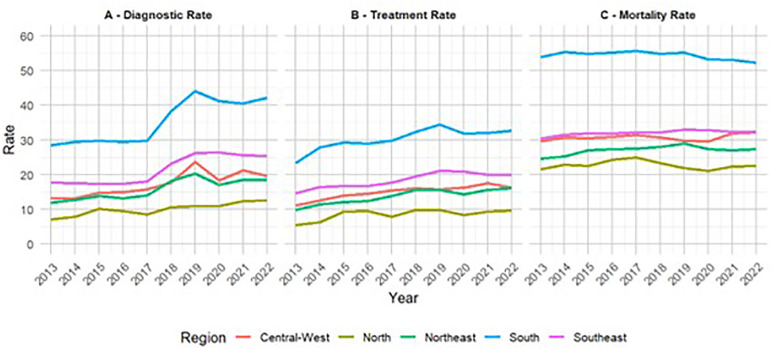
Smoothed age-adjusted (A) diagnostic rate, (B) treatment rate, and (C) mortality rate due to respiratory tract cancers per 100,000 population among 40- to 79-year-old individuals in 2013–2022 in Brazil, stratified by administrative region.

Diagnosis rates ranged from 20.55 to 27.96 per 100,000 population, whereas treatment rates ranged from 17.79 to 23.41 per 100,000 population. Mortality rates exhibited a smaller variation, with a range of 37.25 to 39.12 per 100,000 population. Overall, the rates peaked in 2019. However, in analyzing per administrative region, it was found that the peak in diagnosis occurred in 2022 in the North and Northeast, the peak in treatments occurred in 2021 in the Central-West, and the peak in mortality occurred in 2017 and 2022 in the North and Central-West, respectively (Fig 2).

Temporal analysis of smoothed rates revealed increasing trends in diagnosis and treatment between 2013 and 2022, with visible peaks in 2019 and declines in 2020, followed by recovery in most of the regions. The Mann–Kendall test confirmed the statistical significance (*p* < 0.05) of these trends in most regions, especially in the Southeast and South, which consistently presented the highest diagnosis and treatment rates. Mortality rates were more stable, with a slight but significant increase in the Northeast. The disruptions in 2020 reflect the impacts of COVID-19. It is worth noting that the South exhibited the highest mortality rates in recent years, despite also showing elevated diagnosis and treatment rates. This pattern may reflect a combination of factors, such as higher disease burden, demographic characteristics (e.g., older population), and greater accuracy in registering the causes of death, rather than shortcomings in healthcare access or quality (Fig 2).

Analysis by administrative region revealed significant increases in diagnosis rates in all regions; in treatment rates in the Central-West, Northeast, South, and Southeast; and in mortality rates in the Southeast. These findings suggest important regional variations in both diagnostic capacity and access to treatment. Mortality may be associated with factors such as stage of the disease at diagnosis and available resources (Table 1).

**Table 1:** Mann–Kendall trend analysis of smoothed age-adjusted rates of diagnosis, treatment, and mortality due to respiratory tract cancers per 100,000 population among 40- to 79-year-old individuals in 2013–2022 in Brazil, stratified by administrative region.

	Diagnostic		Treatment		Death	
	t	p	t	p	t	p
**Central west**	0.82	<0.01	0.90	<0.01	0.32	0.24
**North**	0.85	<0.01	0.49	0.06	0.09	0.79
**Northeast**	0.79	<0.01	0.81	<0.01	0.41	0.12
**South**	0.76	<0.01	0.73	<0.01	−0.41	0.13
**Southeast**	0.54	0.04	0.72	<0.01	0.76	<0.01

t= Mann-Kendall tendency; p= p value.

### Analysis of spatial patterns

**Fig 3:**
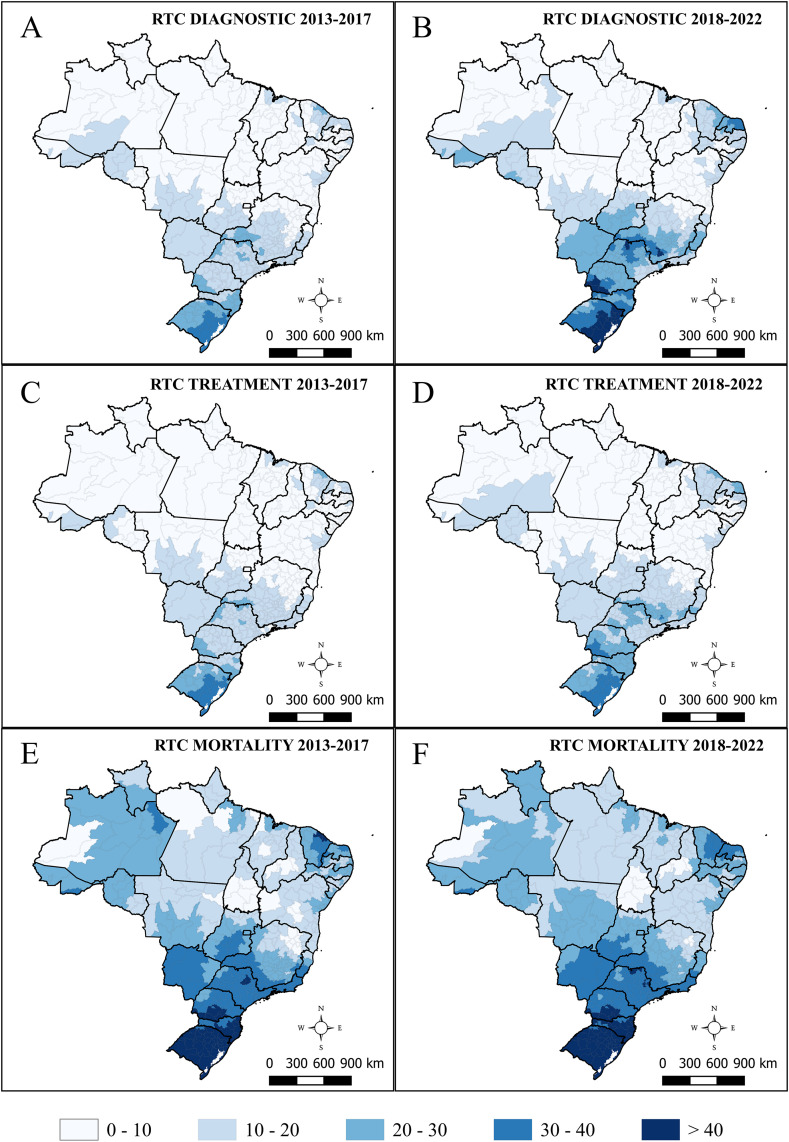
Smoothed age-adjusted rates of respiratory tract cancers per 100,000 population among individuals aged 40–79 years in Brazil, stratified by administrative region. **(A)** Diagnosis rates from 2013 to 2017. **(B)** Diagnosis rates from 2018 to 2022. **(C)** Treatment rates from 2013 to 2017. **(D)** Treatment rates from 2018 to 2022. **(E)** Mortality rates from 2013 to 2017. **(F)** Mortality rates from 2018 to 2022.

Among health regions, between 2013 and 2017, the smoothed rates of diagnosis ranged from 0.00 to 37.54 per 100,000 population, with a predominance of higher rates in the South (Fig 3A). In the following quinquennium (2018–2022), these rates increased to 5.99 to 50.10 per 100,000 population, expanding toward the Southeast, Central-West, and Northeast (Fig 3B). Significant differences were observed in average diagnosis rates across health regions between 2013–2017 and 2018–2022 (*p* < 0.001).

Smoothed age-adjusted treatment rates in 2013–2017 ranged from 0 to 36.13 per 100,000 population, with a predominance of high rates in the South (Fig 3C). In 2018–2022, treatment rates increased to 4.87 to 41.09 per 100,000 population and expanded toward the Southeast (Fig 3D), with significant differences between two periods (*p* < 0.001).

Smoothed age-adjusted mortality rates for the 2013–2017 period ranged from 0 to 77.17 per 100,000 population, with a predominance in the South and high rates in the other regions (Fig 3E). From 2018 to 2022, mortality rates ranged from 17.10 to 77.94 per 100,000 population, with expansion toward the northern region of the Central-West and part of the Northeast (Fig 3F) and significant differences between two periods (*p* < 0.001).

**Fig 4:**
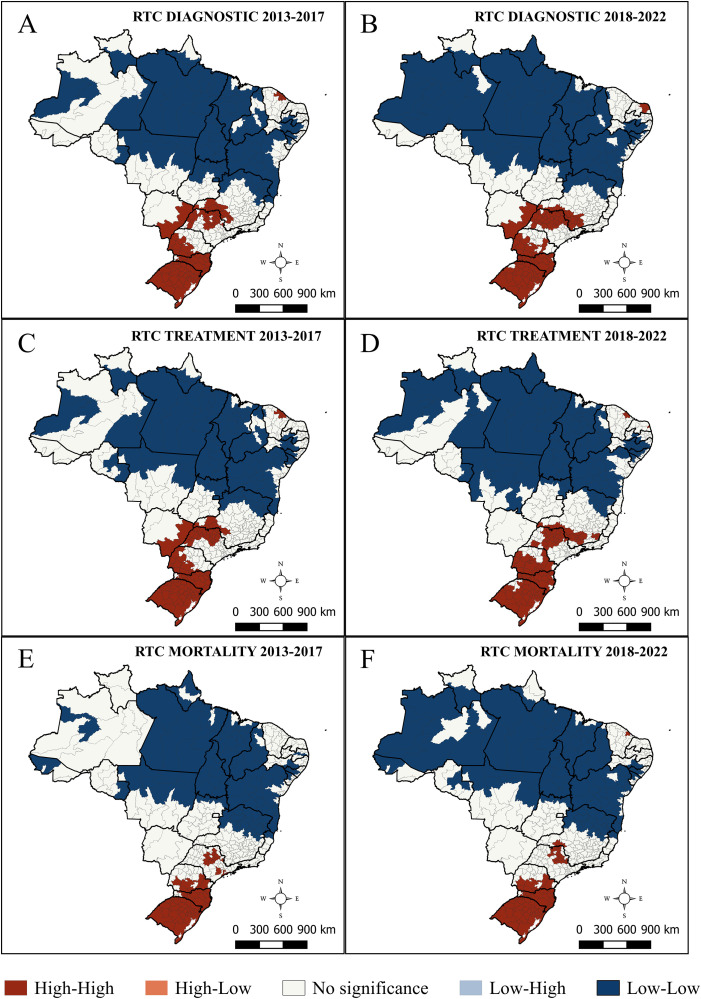
Local indicators of spatial association (LISA) for age-adjusted rates of respiratory tract cancers per 100,000 population among individuals aged 40–79 years in Brazil, stratified by administrative region. **(A)** Diagnosis rates from 2013 to 2017. **(B)** Diagnosis rates from 2018 to 2022. **(C)** Treatment rates from 2013 to 2017. **(D)** Treatment rates from 2018 to 2022. **(E)** Mortality rates from 2013 to 2017. **(F)** Mortality rates from 2018 to 2022.

Spatial autocorrelation analysis of diagnosis rates in the 2013–2017 period revealed a strong spatial dependence, with a Moran’s *I* of 0.889 and **p* *< 0.01. High–high clusters were identified in the South, Central-West, and Southeast, whereas low–low clusters were observed in the North and Northeast (Fig 4A). This pattern persisted in the following quinquennium (2018–2022), with a Moran’s *I* of 0.909 and *p* < 0.01 (Fig 4B).

Treatment rates had a strong spatial autocorrelation in 2013–2017 (Fig 4C), with a Moran’s *I* of 0.893 and **p* *< 0.01. High–high clusters were predominant in the South, Central-West, and Southeast, and low–low clusters in the North and Northeast. In 2018–2022, the pattern was similar (Moran’s *I* = 0.897, *p* < 0.01), except in the Central-West, which did not exhibit high–high clusters (Fig 4D).

In 2013–2017, mortality rates exhibited a strong spatial autocorrelation (Moran’s *I* = 0.928, *p* < 0.01), with high–high clusters in the South and part of the Southeast, and low–low clusters in the Northeast and North (Fig 4E). In 2018–2022, the autocorrelation remained strong, with a Moran’s *I* of 0.937 and *p* < 0.01. The same pattern of high–high clusters was maintained in the South and Southeast. Low–low clusters were concentrated in the Northeast and North (Fig 4F).

**Fig 5:**
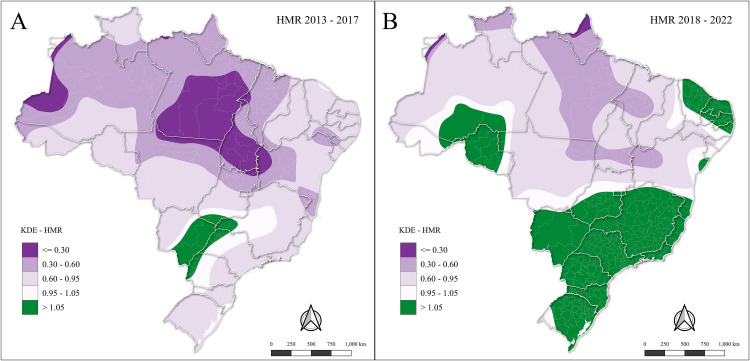
Spatial relative healthcare and mortality ratio (HMR) of respiratory tract cancers between (A) 2013–2017 and (B) 2018–2022 in Brazil, stratified by health region.

Following local spatial autocorrelation analysis (Fig 4), the spatial distribution of HMR based on weighted KDE was generated (Fig 5), comparing diagnosis and treatment events relative to mortality from RTCs.

In 2013–2017 (Fig 5A), higher HMR values (in green) showed only in limited areas concentrated in northern Parana state, the southwestern of Mato Grosso do Sul state, the southern portion of Goias state, and western area of Minas Gerais. Most of the country remained in low HMR, reflecting an imbalance between healthcare availability and mortality. In the 2018–2022 period (Fig 5B), the spatial distribution of high HMR values expanded, covering almost the entire South region (Rio Grande do Sul, Santa Catarina, and Parana states), as well as large parts of the Southeast (São Paulo, Rio de Janeiro, Espírito Santo, and Minas Gerais states) and areas of the Center-West (Mato Grosso and Goias states). Additional green spots also appeared in the Northeast (Bahia, Sergipe, and Alagoa states) and in the North (Amapa, Rondonia, and Para states), indicating a nationwide improvement in HMR, albeit unevenly distributed across regions.

Values close to 1 (white areas in Fig 5A and 5B) represent approximate equivalence between healthcare service density and mortality density. Spatially, these transition zones were mostly located in parts of the Center-West, the interior of the Southeast, the Northeast, and the Acre region, indicating intermediate conditions where expanded diagnostic and treatment services coexist with persistently high mortality.

## Discussion

Despite advances in cancer care, RTCs remain a major global health challenge and one of the leading causes of cancer-related morbidity and mortality. In Brazil, where healthcare access is marked by deep regional inequalities, understanding the spatial and temporal dynamics of RTC outcomes is critical. Our findings highlight persistent geographic disparities in access to diagnostic and treatment services, with mortality rates remaining relatively stable despite a marked rise in the volume of cases diagnosed and treated from 2013 to 2022. These results suggest that improvements in oncological care delivery alone have not translated into better outcomes, particularly in underserved regions [11].

The stability of RTC mortality may partly reflect delays in diagnosis and treatment, which are known to worsen prognosis in RTCs [40–41]. Early-stage detection is associated with significantly better outcomes, as cancers localized at the time of diagnosis are more amenable to curative treatments, such as surgery and targeted therapy [42]. However, barriers such as limited screening, insufficient biomarker testing, and delayed referrals persist, especially in socioeconomically vulnerable populations, limiting timely diagnosis and treatment access [43].

Spatial analysis revealed intensifying hot spots of diagnosis and treatment in the South and Southeast, which may reflect better service availability or higher disease burden in these regions. In this context, the South consistently exhibited higher rates across all indicators. These patterns may reflect greater access to healthcare, as well as wider availability of specialized cancer services in this region [44–45], which facilitate earlier diagnosis and treatment. At the same time, the higher rates might also suggest a greater capacity to detect and record cases, rather than necessarily reflecting a higher underlying disease burden [46]. Moreover, genetic susceptibility and regional lifestyle factors – including tobacco use, alcohol consumption, and dietary patterns – may contribute to these differences [47–48].

Regional disparities in healthcare infrastructure significantly contribute to the observed patterns in respiratory tract cancer (RTC) outcomes across Brazil. According to the 2020 Medical Demographics Report by the Federal Council of Medicine (CFM), physician density varies substantially across the country. The North region has 1.73 physicians per 1,000 inhabitants, while the Southeast has 3.76, the South has 3.27, the Central-West has 3.39, and the Northeast has 2.22 [49]. This uneven distribution affects access to timely diagnosis and treatment, particularly in underserved areas.

Specialized oncology services are also concentrated in certain states. In 2020, approximately two-thirds of the 386 High-Complexity Oncology Centers (CACON/UNACON) and 267 radiotherapy centers were located in Sao Paulo, Rio de Janeiro, Minas Gerais, Parana, and Rio Grande do Sul [50]. This concentration results in limited access to specialized care in regions such as the North and Northeast, leading to delayed diagnoses, reduced treatment options, and higher mortality rates. These structural inequalities underscore the importance of targeted public policies to address regional disparities and improve the efficiency of cancer control strategies.

Surrounding areas of intensifying hot spots showed consistent yet fluctuating patterns, suggesting ongoing high case concentrations despite annual variations. Tumor histology, socioeconomic conditions, and health system capacity likely influence these patterns, with outcomes varying by region [43,51–54]. Even in higher-income regions, many cases are still diagnosed at advanced stages, underscoring the complex interplay between access, quality of care, and patient outcomes.

It is important to note that spatial patterns may differ for other cancer types, such as breast or colorectal cancer, due to differences in risk factors, screening programs, and healthcare pathways. Similar spatial methods, including space–time cubes, Moran’s I, and LISA, have been applied to other cancers, revealing distinct regional clusters of diagnosis, treatment, and mortality [12,55,56]. This comparison highlights that, although our findings are specific to RTCs, the methodological approach is broadly applicable and situates these results within a broader corpus of cancer research addressing spatial inequalities.

In contrast, cold spots in the North and Northeast suggest substantial underdiagnosis and limited access to care, likely driven by geographic isolation, low healthcare infrastructure, and socioeconomic barriers [57–61]. Some areas in the Northeast exhibited oscillating hot spots, indicating inconsistent or fragmented responses from local health systems. These findings underscore the importance of using spatial epidemiology to support targeted public policies aimed at addressing regional inequalities and improving the efficiency of cancer control strategies [2].

Temporal analysis showed a peak in diagnosis, treatment, and mortality in 2019, followed by a sharp decline in 2020–2021 due to the COVID-19 pandemic. This pattern aligns with previous studies reporting reduced healthcare use and delayed cancer diagnoses during the pandemic, exacerbated by hospital overload and fear of infection [62–63]. Notably, lung biopsies dropped by over 90%, and more aggressive tumor types became more frequent during this period, potentially worsening mortality outcomes [64–65].

Consistently high rates of diagnosis, treatment, and mortality were observed in the South. Previous studies also identified high–high mortality clusters of cancer in the South and Central-West [9,67], whereas mortality patterns in other regions were more dispersed and variable over time. Although diagnosis and treatment expanded in recent years, they failed to shift mortality trends, likely due to late-stage presentation and continued barriers to early detection. In Brazil, over 85% of lung cancers are diagnosed at advanced stages, especially in low-income populations [65–66].

Despite the increase in service capacity, access to cancer care in Brazil remains unequal. In 2013–2017, high HMR values were limited to specific and limited areas in the South of Brazil, while most regions showed low-to-intermediate ratios. In the areas with high HMR, there was an increase in the diagnostic services, reflecting persistent disparities between healthcare availability and mortality. In the next quinquennia, HMR improved across almost all the South and Southeast regions and expanded into parts of Center-West, North, and Northeast, indicating nationwide progress in healthcare relative to mortality. Even with the improvement in the HMR, some regions still lagged behind due to chronic underfunding and logistical challenges [11]. These disparities raise concerns about the equity and effectiveness of Brazil’s cancer control policies and highlight the need for targeted interventions to ensure timely access to care and reduce regional inequalities.

From a global health standpoint, these findings have implications for Brazil’s progress toward meeting the Sustainable Development Goals (SDGs), particularly SDG 3 (Good Health and Well-being) and SDG 10 (Reduced Inequalities). Although mortality has slightly declined among men, possibly due to tobacco control policies, rates remain stagnant or rising among women and in underserved regions [65–67].

Our study has some limitations, including the use of aggregated secondary data, which may mask individual-level variations and does not allow analysis by tumor histology or staging. Furthermore, delays between diagnosis and treatment could not be evaluated directly, and underreporting in administrative databases, especially in the North and Northeast, may lead to burden underestimation in these regions. Despite these constraints, the study offers robust spatiotemporal evidence to guide regionally tailored cancer control strategies and strengthening efforts for the health system.

In addition to underreporting and deaths among individuals who never access healthcare services, other factors may also contribute to the observed disparities. These include delays in diagnosis, variation in the quality of death certificate completion (with higher proportions of ill-defined causes in some regions), and patient migration to referral centers, a phenomenon that may lead to misclassification of the geographic location of death. Moreover, differences in diagnostic infrastructure, such as pathology and imaging capacity, may lead to cancer cases being recorded as unspecified neoplasms. Together, these limitations underscore the role of data quality and health system heterogeneity in shaping spatial cancer patterns in Brazil [68–70].

Our findings demonstrated spatiotemporal variation in RTC diagnosis and treatment, whereas mortality showed only spatial variation. This information can help managers develop strategies focused on mortality hot spots. The present study does not directly model temporal delays between diagnosis, treatment, and mortality; however, it is important to acknowledge that such lags are highly plausible in real-world oncology care. For instance, an increase in diagnostic efforts or access to care in a given year, such as 2018, may only translate into measurable changes in mortality trends in subsequent years.

## Conclusion

The findings demonstrated an increasing trend in RTC diagnosis and treatment. However, mortality rates have remained stable over the years, with the South consistently exhibiting higher rates. This suggests that improvements in diagnostic and treatment services have occurred but remain insufficient to reduce RTC mortality at the national level. Notably, the spatial distribution of KDE values revealed consistently more effective healthcare coverage in the South and Southeast regions across both periods, with clear expansion into parts of the Central-West and Northeast hinterland in recent years. However, the North and some areas in the Northeast continue to face significant disparities. Although there has been progress in expanding service delivery beyond major urban centers, these efforts have not yet translated into a uniform or widespread reduction in mortality rates.
